# Efficacy and safety of Apixaban in the treatment of cerebral venous sinus thrombosis: a multi-center study

**DOI:** 10.3389/fneur.2024.1404099

**Published:** 2024-05-16

**Authors:** Naaem Simaan, Issa Metanis, Asaf Honig, Hen Hallevi, Andrei Filioglo, Rom Mendel, Rani Barnea, Jonathan Naftali, Eitan Auriel, Shorooq Aladdin, David Orion, Najib Dally, Ronen R. Leker, Jeremy Molad

**Affiliations:** ^1^Department of Neurology, Ziv Medical Center, Safed, Israel; ^2^The Azrieli Faculty of Medicine, Safed Bar Ilan University, Safed, Israel; ^3^Hadassah Departments of Neurology, Hebrew University Medical Center, Jerusalem, Israel; ^4^Department of Neurology and Stroke, Tel-Aviv Sourasky Medical Center, Tel-Aviv, Israel; ^5^Faculty of Medicine and Sagol School of Neuroscience, Tel-Aviv University, Tel-Aviv, Israel; ^6^Department of Neurology, Assuta Ashdod Medical Center, Ashdod, Israel; ^7^Department of Neurology, Rabin Medical Center, Petah Tikva, Israel; ^8^Departments of Neurology, Sheba Medical Center, Ramat Gan, Israel; ^9^Department of Hematology, Ziv Medical Center, Safed, Israel

**Keywords:** Apixaban, cerebral sinus and venous thrombosis (CSVT), stroke, direct oral anticoagulants (DOAC), vitamin K antagonists (VKA)

## Abstract

**Background:**

Information regarding the safety and efficacy of specific direct oral anticoagulants (DOAC) in the treatment of cerebral sinus and venous thrombosis (CSVT) is scarce. Apixaban is one of the most frequently prescribed DOACs. Therefore, we aimed to compare the safety and efficacy of Apixaban with those of vitamin k antagonists (VKA) in patients with CSVT.

**Methods:**

Prospective CSVT databases from seven academic medical centers were retrospectively analyzed. Patients treated with Apixaban were compared to those treated with VKA. Data on demographics, clinical presentations, risk factors, radiological and outcome parameters were studied.

**Results:**

Overall, 403 patients were included in the analysis. Of them, 48 (12%) were treated with Apixaban, and 355 (88%) were treated with VKA. Rates of coagulopathies were significantly higher in the VKA-treated patients but no other differences between the groups were found in baseline characteristics and underlying etiology. No significant differences were found between groups in efficacy or safety parameters including the rates of recanalization, favorable outcomes, one-year mortality, seizures, intracranial hemorrhage or CSVT recurrences.

**Conclusion:**

Our data suggests that Apixaban may be safe and effective for patients with CSVT. These results should be tested in prospective randomized clinical studies.

## Introduction

Cerebral sinus and venous thrombosis (CSVT) represent 0.5–1% of all stroke cases with an annual incidence of approximately 15 per million (1, 2). CSVT frequently affects young people (3–5). Current guidelines recommend using low-molecular-weight heparin (LMWH) in the acute phase of CSVT, followed by vitamin K antagonists (VKA) (2, 6). Importantly, there are no current recommendations for use of direct oral anticoagulants (DOAC) for CSVT. This is likely due to the existence of only limited data regarding their safety and efficacy in CSVT (7–9). Only two randomized controlled trials examining the safety and efficacy of DOACs in patients with CSVT were published to date, however both included only a limited number of patients precluding concrete recommendations and change in guidelines (10, 11).

Apixaban is currently believed to be the most frequently prescribed DOAC among patients with atrial fibrillation, with previous trials describing excellent safety and efficacy profiles which may be superior to VKA and other DOACs (12, 13). However, little is known about its safety and efficacy profiles in patients with CSVT, with only few case reports and small case series published to date (14–17). Therefore, we aimed to examine the safety and efficacy of Apixaban compared to VKA in the treatment of CSVT patients included in a large multi-center observational cohort.

## Methods

### The dataset

Data from the Israeli CSVT study was retrospectively analyzed. This is an ongoing observational study, continuously enrolling adult patients diagnosed with non-traumatic CSVT that were admitted to seven comprehensive stroke centers in Israel (18). The study was approved by the ethical committees in each of the participating centers.

**Figure 1:**
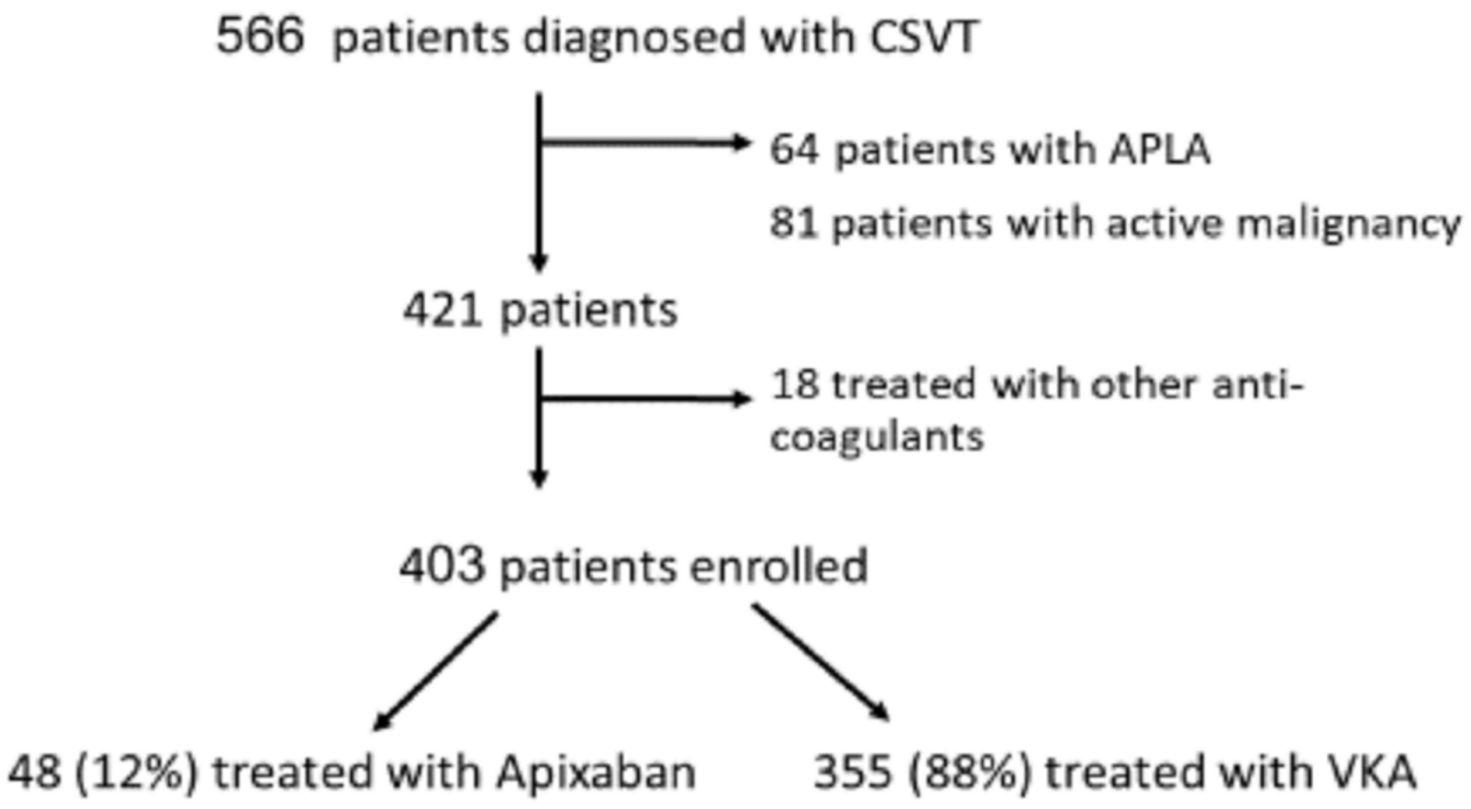
Flowchart of patient inclusion and distribution in the study.

The current analysis included patients diagnosed with CVST between January 2010 and December 2022. Patients with active malignancy or anti-phospholipid syndrome (APLA) were excluded from the current analysis because these patients were treated solely with VKA in our cohort as published before (9). We also excluded patients treated with other DOACs (Figure 1). None of the patients had rheumatic heart disease, valvular atrial fibrillation or mechanical heart valve.

### CSVT diagnosis

The diagnosis of CSVT was based on CT venography or MR venography. All imaging studies were interpreted by experienced neuroradiologists and experienced stroke neurologists. The site and location of the occluded venous were recorded as previously described (9, 18). Multiple vein/sinus involvement was defined as involvement of more than one site, excluding continuous involvement of the transverse and sigmoid sinuses on the same side or cortical vein involvement. The presence of intracerebral hemorrhage (ICH) or venous infarcts were also recorded on admission or follow-up imaging.

Data regarding patient demographics, possible etiologies, comorbidities, vascular risk factors and clinical presentations were documented. Patients underwent a thorough investigation for the cause of CSVT as previously published (19).

### Treatment

In all participating centers, patients were started on LMWH in the acute phase (usually 5–10 days after diagnosis), followed by VKA or Apixaban. Treatment decisions at the individual patient level were taken by the vascular neurology team in each of the participating centers at the discretion of the attending stroke neurologist. Documentation of the reasons for choosing specific treatments in each individual case was not available.

Patients were followed-up at the participating centers’ outpatient stroke clinics after the index event. Occurrence of early (starting during the acute admission) and late (starting after more than 30 days from onset) seizures was documented. Determination of recanalization was documented on follow up CT venography or MR venography. Outcome was measured with the modified Rankin Scale obtained at 90 days (mRS-90) and favorable outcome was defined as an mRS ≤ 2. Mortality was documented from the medical records after 1 year.

### Statistical analysis

Statistical analysis was performed using SPSS software (version 29.0, IBM, Chicago, IL, United States). Continuous variables were reported as a mean ± standard deviation or median and interquartile range (IQR), and dichotomous variables were reported as percentage of the total. Student’s t-test was used for comparisons between categories of continuous variables, chi square test for comparisons of qualitative variables, and the Mann–Whitney test for comparisons of non-parametric variables. A *p*-value ≤ 0.05 was considered statistically significant.

## Results

The entire cohort included 566 CSVT patients. We excluded 64 patients with antiphospholipid syndrome (APS) and 81 patients with underlying active malignancy due to the limited data regarding the safety and efficacy of DOAC treatment or their relative ineffectiveness in these specific conditions (20–23). We also excluded 18 patients treated with other DOACs (Figure 1). The remaining 403 patients were included in the current study (mean age 41 ± 18 years, males = 33%). Data regarding mRS-90 and mortality was available for 96% and 98% of patients, respectively.

Among the 403 patients, 48 (12%) were treated with Apixaban and 355 (88%) were treated with VKA (Table 1). No significant differences were found between the groups in age, sex and other potential risk factors for underlying etiologies of venous thromboembolism, other than factor V deficiency. All patients with factor V deficiency were treated with VKA (10% vs. 0%, *p* = 0.024). In addition, patients with any underlying laboratory detected coagulopathy (Table 1) were more likely to be treated with VKA (35% vs. 12.5%, *p* = 0.002). The presence of other hematological disorders did not differ between the VKA and Apixaban-treated groups. Headache as a presenting symptom was more common in the Apixaban-treated group (90% vs. 76%, *p* = 0.019), but other signs or symptoms suggestive of increased intracranial pressure did not differ between the groups. No differences were found between the groups in the rates of seizures or focal neurological deficits.

**Table 1:** Clinical and radiological characteristics of patients with CSVT.

	Apixaban	VKA	*p*
	*N* = 48	*N* = 355	
Age, mean (SD)	41.5 (18.8)	41.1 (18.0)	0.844
Gender, male (%)	16 (33.3)	120 (33.8)	0.917
Dehydration (%)	1 (2.1)	10 (2.8)	0.574
Neurosurgery (%)	1 (2.1)	9 (2.5)	0.674
Epidural (%)	0 (0)	9 (2.5)	0.265
Infections (%)	1 (2.1)	32 (9.0)	0.100
Smoking (%)	13 (27.1)	74 (20.8)	0.350
Hyperlipidemia (%)	5 (10.4)	54 (15.2)	0.69
Hypertension (%)	6 (12.5)	49 (13.8)	0.773
Obesity (%)	3 (6.3)	32 (9.0)	0.239
Diabetes (%)	4 (8.3)	32 (9.0)	0.814
Past malignancy (%)	2 (4.2)	4 (1.1)	0.103
Previous thrombotic events (%)	4 (8.3)	28 (7.9)	0.919
Pregnancy/post-partum	3 (6.3)	29 (8.2)	0.644
IVF	2 (4.2)	6 (1.7)	0.248
Oral contraceptives (%)	18 (37.5)	91 (25.6)	0.094
**Clinical presentation**			
Papilledema (%)	13 (27.1)	94 (27.7)	0.992
Headache (%)	43 (89.6)	269 (75.6)	**0.019**
Vomiting (%)	7 (14.6)	57 (16.1)	0.349
Seizure (%)	11 (22.9)	74 (20.8)	0.749
Any focal neurological deficit (%)	12 (25.0)	86 (24.2)	0.923
NIHSS admission, (IQR)	0 (0–0)	0 (0–0)	0.117
**Hematological workup**			
Protein C/S deficiency (%)	1 (2.1)	20 (6.0)	0.537
Factor V deficiency (%)	0 (0)	34 (10.3)	**0.024**
Factor II mutation (%)	0 (0)	9 (2.8)	0.265
PT 20210	2 (4.2)	19 (5.3)	0.707
Behcet’s disease (%)	1 (2.1)	19 (5.3)	0.718
MTHFR (%)	3 (6.3)	18 (5.1)	0.763
JAK 2 (%)	1 (2.1)	29 (8.2)	0.086
Thrombocytosis (%)	1 (2.1)	22 (6.2)	0.255
Hyperhomocisteinemia (%)	0 (0)	6 (1.7)	0.36
Any coagulopathy (%)	6 (12.5)	124 (34.9)	**0.002**
**Radiological findings**			
Multiple veins (%)	16 (33.3)	91 (25.6)	0.267
Cortical (%)	8 (16.7)	44 (12.4)	0.421
Deep (%)	4 (8.3)	13 (3.7)	0.135
Venous infarction (%)	4 (8.3)	48 (13.5)	0.262
ICH (%)	8 (16.7)	66 (18.6)	0.734
Any recanalization (%)	211 (86.5)	33 (80.5)	0.312
Complete recanalization (%)	119 (48.8)	17 (41.5)	0.386
**Involved sinus (%)**			
Superior sagittal sinus (%)	13 (27.1)	138 (38.9)	0.104
Transverse sinus (%)	40 (83.3)	245 (69.0)	**0.049**
Sigmoid sinus (%)	30 (62.5)	231 (65.1)	0.670
Cavernous sinus (%)	0 (0)	7 (2.0)	0.324

VKA, vitamin K antagonists; IVF, in-vitro fertilization; NIHSS, *National Institutes of Health Stroke Scale*; IQR, interquartile Range; PT 20210, Prothrombin 20210; MTHFR, methylenetetrahydrofolate reductase; JAK2, Janus kinase 2; ICH, intracerebral hemorrhage. *p* values in bold are statistically significant.

Patients with transverse sinus involvement were more likely to be treated with Apixaban (83% vs. 69%, *p* = 0.049). No differences were found between the groups in the involvement of other venous sinuses, rates of multiple veins involvement, cortical vein involvement, deep vein involvement, venous infarction and ICH.

No differences were found between groups in the rates of outcome measurements including the rates of any recanalization and complete recanalization, favorable outcome at 90 days, mortality within 1 year and recurrent CSVT (Table 2). Sub-analysis excluding patients with underlying coagulopathy demonstrated similar results.

**Table 2:** Outcomes in patients with CSVT treated with Apixaban or VKA.

Outcome	Apixaban *N* = 48	VKA N = 355	*P*
mRS 90 (IQR)	0 (0–1)	0 (0–1)	0.708
Favorable outcome (%)	45 (93.8)	304 (88.9)	0.304
Mortality (%)	0 (0)	7 (2.0)	0.319
Recurrent CSVT (%)	0 (0)	10 (3.3)	0.230

mRS, Modified Rankin Scale; CSVT, Cerebral Venous Sinus Thrombosis.

## Discussion

The current multi-center study demonstrated that Apixaban may be a safe and effective alternative to VKA for the treatment of CSVT patients. Apixaban was associated with similar rates of favorable outcomes, mortality, recanalization and risk of hemorrhage compared to VKA.

Of note, patients with coagulopathies were more likely to be treated with VKA. These results probably reflect the limited data regarding the safety and efficacy of DOACs in patients diagnosed with CSVT and underlying coagulopathies directing treatment decisions toward choosing VKA. Our findings are in line with the results reported in previous studies focusing on DOAC treatment for CSVT (10, 24–27). our results are also in concordance with the results of previous studies specifically showing the safety and efficacy of Apixaban in patients with CSVT. However, these previous case series only included very few patients treated with Apixaban (14–17, 26). Therefore; the current results offer further reassurance regarding the efficacy and safety of Apixaban in patients with CSVT.

It should be noted that although no significant differences in safety or efficacy were found between the treatment groups in the current study, a non-significant trend favoring Apixaban was found in all clinical outcome parameters, including higher rates of favorable outcome, lower mortality rates and lower recurrence rates. This consistent trend may potentially point to the possibility of superiority of Apixaban treatment over VKA among CSVT patients, which may have been missed in the current study due to lack of statistical power. This possibility should be further examined in future larger trials.

Over the last decade, DOACs became the treatment of choice for atrial fibrillation and deep venous thrombosis due it clear benefits over VKA (8, 28). The greatest proportional increase in use over time among DOACs was observed with Apixaban (29, 30). This is probably because of its favorable safety profile compared with Dabigatran and Rivaroxaban (31–33). Apixaban was also compared with Aspirin in atrial fibrillation patients who were unable or unwilling to take VKA. In these studies, it was found to reduce the risk of stroke and systemic embolism compared to aspirin with similar rates of major bleeding (12, 34).

A meta-analysis focusing on the effects of DOACs in CSVT showed that DOACs are as safe and effective as VKA (31). Furthermore, a large retrospective observational study that included 845 CSVT patients and compared DOAC with warfarin, found a lower risk of ICH in the DOAC treated group as well as similar rates of recurrent VTE, death, and recanalization (27). These studies did not compare between different DOACs. Two randomized controlled studies that explored the efficacy of DOACs in patients with CSVT were published thus far (10, 11). The first study, found that Dabigatran has similar safety and efficacy profiles compared to those of VKA (10). The second study, which included only 55 patients randomized to receive Rivaroxaban or VKA demonstrated higher but non-significant rates of ICH and recurrent CSVT among patients treated with Rivaroxaban (11). Currently, another large ongoing observational non-randomized study focusing on the role of DOAC vs. VKA in CSVT patients (DOAC-CVT study) is still enrolling patients (35). Taken together, the results of these and other future trials may better establish the safety and efficacy profiles of DOACs in patients with CSVT and help shed more light on whether these effects represent a class effect or whether one DOAC is superior to the others.

The strengths of the current analysis include the relatively large cohort size and high follow-up rates. Study limitations include the retrospective design of this analysis which is susceptible to possible bias. Second, we did not have data regarding the reasons for the type of treatment chosen. However, it should be noted that no difference in baseline characteristics or risk factor was found between groups other than the rates of factor V deficiency or any coagulopathy, and that a sub-analysis excluding such patients demonstrated similar results. Third, we could not compare Apixaban with other DOACs, as only 18 patients in our registry were treated with Dabigatran or Rivaroxaban. Fourth, we did not compare different doses of Apixaban or analyze the amount of time spent in the therapeutic range among patients treated with VKA or Apixaban. Fourth, data regarding recanalization was only available for 301 patients (75%), as some follow-up imaging were not performed at the participating centers. Lastly, the clinical outcome measures were not assessed blinded to the treatment modality.

In conclusion, our data suggests that Apixaban seems to be as safe and effective as VKA in patients with CSVT without underlying APLA or active malignancies.

## Data availability statement

The original contributions presented in the study are included in the article/supplementary material, further inquiries can be directed to the corresponding author.

## Author contributions

NS: Conceptualization, Data curation, Formal analysis, Funding acquisition, Investigation, Methodology, Project administration, Resources, Software, Supervision, Validation, Visualization, Writing – original draft, Writing – review & editing. IM: Conceptualization, Investigation, Software, Writing – original draft. AH: Conceptualization, Methodology, Writing – review & editing. HH: Conceptualization, Supervision, Validation, Writing – review & editing. AF: Data curation, Formal analysis, Writing – review & editing. RM: Software, Supervision, Validation, Writing – review & editing. RB: Investigation, Methodology, Project administration, Writing – review & editing. JN: Methodology, Project administration, Writing – review & editing. EA: Methodology, Software, Writing – review & editing. SA: Investigation, Project administration, Validation, Writing – review & editing. DO: Investigation, Methodology, Project administration, Writing – review & editing. ND: Methodology, Project administration, Resources, Writing – review & editing. RL: Data curation, Formal analysis, Funding acquisition, Writing – review & editing. JM: Conceptualization, Data curation, Formal analysis, Funding acquisition, Investigation, Methodology, Project administration, Resources, Software, Supervision, Validation, Visualization, Writing – original draft, Writing – review & editing.
